# Continued Organic Fertigation after Basal Manure Application Does Not Impact Soil Fungal Communities, Tomato Yield or Soil Fertility

**DOI:** 10.3390/microorganisms11071715

**Published:** 2023-06-30

**Authors:** Jose Ignacio Marín-Guirao, María del Carmen García-García, Emilio Martín-Expósito, Miguel de Cara-García

**Affiliations:** Andalusian Institute of Agricultural and Fisheries Research Training (IFAPA) La Mojonera, Camino San Nicolás, 1, 04745 Almería, Spain; mariac.garcia.g@juntadeandalucia.es (M.d.C.G.-G.); emilio.martin.exposito@juntadeandalucia.es (E.M.-E.)

**Keywords:** agroecological soil modelling, culturable fungi, DNA metabarcoding, greenhouse, ITS2 genomic region, organic farming, plate culture method

## Abstract

There is currently a limited understanding of the complex response of fungal microbiota diversity to organic fertigation. In this work, a 2-year field trial with organic tomato crops in a soil previously amended with fresh sheep manure was conducted. Two hypotheses were compared: (i) fertigation with organic liquid fertilizers versus (ii) irrigation with water. At the end of both years, soils were analyzed for physical–chemical parameters and mycobiome variables. Plate culture and DNA metabarcoding methods were performed in order to obtain a detailed understanding of soil fungal communities. Fertigation did not increase any of the physical–chemical parameters. Concerning soil fungal communities, differences were only found regarding the identification of biomarkers. The class *Leotiomycetes* and the family *Myxotrichaceae* were identified as biomarkers in the soil fungal community analyzed by means of DNA metabarcoding of the “fertigation” treatment at the end of Year 1. The *Mortierella* genus was detected as a biomarker in the “water” treatment, and *Mucor* was identified in the “fertigation” treatment in the cultivable soil fungi at the end of Year 2. In both years, tomato yield and fruit quality did not consistently differ between treatments, despite the high cost of the fertilizers added through fertigation.

## 1. Introduction

Improving soil health and reducing agricultural inputs are crucial factors in ensuring sustainable and profitable farming practices. These are key elements to reversing the degradation of agrosystems and, at the same time, to addressing the substantial threat of climate change and population growth on a global scale. Hence, the implementation of efficient agricultural techniques aimed at reducing the impacts generated by productive systems in recent decades has emerged as a critical priority on the international political agenda. In this context, organic farming (OF) has been identified as playing a key role in realizing the Sustainable Development Goals outlined in the United Nations’ Agenda 2030, as well as in European Union policies such as the EU Green Deal’s Farm to Fork initiative or those contained in the EU Soil Mission program.

OF has experienced significant growth in recent years, as reflected in the constant increase in certified farming areas in accordance with Regulation (EU) No 2018/848. This development is evident throughout the European Union (EU), where approximately 14.7 million hectares of land assigned to OF was recorded in 2020 (including areas in conversion), representing a 56% increase between 2012 and 2020, and accounting for 9.1% of the total utilized agricultural area (UAA). This increase is also evident in Spain, the EU country with the second-largest area dedicated to OF, after France. In 2020, Spain had over 2.43 million hectares of organic production, representing nearly 10% of the country’s UAA, surpassing the European average [1]. Andalusia, the Spanish autonomous community with the largest area dedicated to organic production within the national territory, had a certified organic greenhouse area of 5561 hectares in 2021. Notably, the province of Almeria (in southeastern Spain), which had 892 hectares of OF in 2010, had 4382 hectares in 2021 (data referring to farms with the last annual visit conducted by the private control agency) [2], reflecting the great interest in the sustainability of vegetable production systems in greenhouses. Almeria, with a total greenhouse area of 32,827 hectares, allocated over 8200 hectares to the production of greenhouse tomatoes in the 2021–2022 campaign. This makes Almería the main winter tomato production area in Europe, and, consequently, the main supplier of fresh tomatoes (organic and non-organic) during the coldest months of the year [3].

According to Regulation (EU) No 2018/848 on organic production, cultivation practices should aim to maintain or increase soil organic matter (SOM), enhance soil stability and soil biodiversity, and prevent soil compaction and soil erosion. To achieve this, soil fertility and biological activity must be maintained and improved through crop rotations that include leguminous crops and green manure crops and by limiting the use of external inputs into livestock manure or organic matter. In addition, fertilizers, soil conditioners, and nutrients listed in accordance with the Commission Implementing Regulation (EU) 2021/1165 on products and substances for use in organic production may only be used to the extent necessary when the nutritional needs of plants cannot be met.

In this regard, two main paths have been established to shift soil modeling from a chemical-based approach to an agroecological biological approach [4]. One path involves solely input substitution, such as replacing mineral fertilizers with microbiological and/or other fertilizers listed in Regulation (EU) 2021/1165. The other path involves a holistic approach to improve soil health, by increasing SOM to enhance soil fertility [5,6], for example by using organic matter amendments. For the second path, which is the most desirable to achieve a real transition to sustainable practices and meet the aforementioned international policies, it is essential to consider soil microorganisms as key players in maintaining and improving the health and fertility of agricultural soils [7,8]. In this regard, fungi play a crucial role in soil ecosystems and are essential for the breakdown of organic matter, nutrient cycling, the formation and stabilization of soil aggregates, and disease suppression [7,9,10,11]. Therefore, both SOM and soil fungi should be considered essential factors in achieving high greenhouse soil fertility, and ultimately ensuring optimum crop development in soils without inorganic fertilization [12]. For these reasons, it is crucial to understand the composition of fungal communities and their response to environmental changes for sustainable soil management. Agricultural management practices, such as tillage, fertilization, and crop rotation, can affect fungal community composition in agroecosystems [13,14,15,16,17,18,19,20,21]. In particular, since fertilization management can affect plant performance and soil microbiota, the impact of fertigation practices on soil fungal diversity and community structure is an important area of study for sustainable greenhouse vegetable production.

Before a greenhouse area can be certified as ‘organic’, it must undergo a conversion process, which commonly takes 2 years for vegetable crops. During this period, the primary challenge in transitioning to the agroecological approach is to restore and/or improve natural soil biodiversity and fertility while maintaining economic profitability to allow most farmers to make the transition. In this regard, it is of great interest to assess the fertilizing potential of manure applied as a basal dressing in the organic production of high-yielding greenhouse vegetable crops, as well as its impact on soil fertility linked to its fungal microbiota. This information helps determine if a manure-based basal dressing is sufficient for an economically viable organic vegetable crop without showing any deficiency compared to a crop that additionally receives a fertigation plan with organic fertilizers. Therefore, the main objective of this study is to evaluate the impact, over two consecutive years, of fertigation with fertilizers listed in Regulation (EU) 2021/1165 in a greenhouse tomato crop and soil after a fresh sheep manure-based basal dressing. To achieve this, the following specific objectives were addressed: (i) to evaluate the physicochemical properties and fungal microbiota of the soil at the beginning of the crop cycle and after both types of management in the two-year study, (ii) to evaluate tomato fruit production and quality for both years, and (iii) to estimate the cost of the fertilizers included in the fertigation plan in the two crop cycles.

## 2. Materials and Methods

### 2.1. Overview of the Location and the Experimental Greenhouse

The trials were conducted for two consecutive years (the 2019/2020 and 2020/2021 seasons) at the Andalusian Institute for Research and Training in Agriculture and Fisheries (IFAPA) in Almería (36°48′ N, 2°41′ W; altitude 142 m), the biggest Mediterranean greenhouse cropping region, and the main organic winter tomato production area in Europe. The climate in the area is Mediterranean arid, characterized by mild winters and hot summers with no rainfall. The experimental greenhouse was representative of the “raspa y amagado” Mediterranean greenhouse, which includes the use of sand mulch, locally known as “arenado”. This involves covering the surface of the crop field with a layer of silica sand [22]. The soil in the greenhouse was classified as sandy loam according to the United States Department of Agriculture (USDA) soil classification. It was a greenhouse which had been certified for organic production by the Andalusian Organic Farming Committee (C.A.A.E.) since 2006. The greenhouse had a west–east orientation, with crops rows aligned north–south, and covered an area of 832 m^2^. The maximum and minimum heights inside the greenhouse were 3.9 and 2.3 m, respectively. The irrigation system was automated, with droppers spaced 0.5 m apart and a discharge rate of 3 L h^−1^. The greenhouse was covered with a 200 µm thick polyethylene material, which has a theoretical transmissivity of 90% and thermal properties. It also had zenithal and lateral ventilation, with an anti-thrip-aphid mesh that had 20 × 10 threads cm^−2^. The greenhouse had four lateral windows and two zenithal windows facing east and west. Similarly, the lateral and zenithal windows had deflectors in their lower part, which improved air circulation in the area occupied by the crop [23].

### 2.2. Plant Material, Cropping Details and Experimental Design

Two subsequent winter cycles of tomato (*Solanum lycopersicum* L.) “Valenciano type” plants, grafted onto Armstrong^®^ rootstock (Syngenta, Basel, Switzerland), were grown in the same greenhouse in the seasons 2019/20 and 2020/21 (i.e., Year 1 and Year 2, respectively). Four-week-old tomato plants were planted on 17 September 2019 and 25 September 2020, in Years 1 and 2, respectively. In both years, prior to planting, in July, fresh sheep manure was buried uniformly across all crop rows in the greenhouse at a rate of 4 kg m^−2^. The incorporation was performed by removing the top layer of sand and mixing the manure with the soil using a rotavator. The area was then covered with the same sand that was previously removed. Next, drip lines were installed and, with the aim of favoring the manure’s decomposition, the soil was covered with transparent polyethylene film (30 µm TIF Desinfección DS^®^, Sotrafa, Almería, Spain) for a period of two months, after a single irrigation application to reach saturation at a 15 cm depth, which is known as the soil biosolarization technique. Planting took place two days after removing the film. Crop growth occurred on two axes, considering each as an individual plant for sampling purposes, thus resulting in an overall density of 2 plants m^−2^. Tomato vines were vertically trained with polypropylene strings and pruned and managed according to established local practices. For correct and optimal pollination, bumblebees (*Bombus terrestris*) were used. Crop management and pest control were guaranteed by adhering to Regulation (EU) 2018/848 on organic production. As there were no soil-borne pathogens present in the soil, no soil treatments were necessary during the crop periods.

The study considered two treatments (Table 1 and Table 2): (i) “water” received only the initial application of manure and was irrigated solely with water throughout the crop cycles and (ii) “fertigation” supplemented the manure with fertilizers listed in the Commission Implementing Regulation (EU) 2021/1165 on products and substances for use in organic production, which were applied through fertigation. The experiment was performed as a single-factor design with three replications (*n* = 3). The trial consisted of a total of 6 experimental plots, each being 96 m^2^ in size.

### 2.3. Soil Sampling

Soil samples were taken at three different sampling times. To assess the starting situation, soil sampling was carried out in three areas of the greenhouse immediately before planting the tomato seedlings. Thus, the soil samples were taken after the biosolarization treatment with fresh sheep manure, two days before planting (i.e., initial, 16 September 2019). Similarly, in both years, at the end of the tomato crop season (i.e., end of Season 1, 3 April 2020, and end of Season 2, 23 April 2021, in Year 1 and Year 2, respectively), all of the experimental plots were sampled. After removing the top layer of sand, the samples were taken with an auger at a depth of 0–30 cm. Three subsamples were randomly taken from cultivation lines and then mixed and homogenized to ensure representativeness in each sample. Subsamples were taken in the center of the cultivation lines to avoid a possible edge effect. For each soil sample, one subsample was frozen at −80 °C for DNA extraction, another subsample was air-dried and sieved (<2 mm) for chemical analysis, and another subsample was air-dried and sieved (<0.2 mm) for microbiological analysis through the plate culture method.

### 2.4. Analysis of Physical-Chemical Characteristics of Soil Samples

Soil samples (*n* = 3) were analyzed for pH, electrical conductivity (EC), interchangeable cations (Ca^+2^; Na^+^; Mg^+2^; K^+^), active limestone, phosphorus Olsen (P Olsen), nitric nitrogen, organic matter, total carbonates (HCO_3_^−^), total nitrogen, and C/N ratio, following standard soil testing procedures as described in Order 5/12/1975 [24] by an external laboratory (Eurofins, El Ejido, Spain). The results for most physical and chemical variables of soil samples were reported in different units for Years 1 and 2. Comparisons among the treatments were made for each year separately.

### 2.5. Study of Soil Fungi in Soil Samples

The study of soil fungal microbiota (“mycobiome”) was conducted using two evaluation methods: (i) the plate culture method (culturable fungal microbiota) and (ii) a molecular method based on DNA metabarcoding.

#### 2.5.1. Assessment of Culturable Fungal Microbiota

- Preparation of soil samples

Soil samples were subjected to a drying, grinding, and sieving process, following Tello et al. [25]. Drying was conducted at room temperature (20–25 °C) for 7–10 days, until soil humidity was constant and homogenous. A porcelain mortar was used for grinding, and a 200 μm mesh-size sieve was used for sieving. The mortar and the sieve were washed and disinfected between samples by covering them with alcohol and lighting it.

- Analytical method

The soil culturable fungal microbiota were analyzed by means of the serial dilution method [25]. The culture medium used was potato dextrose agar (PDA). Five subreplicates (i.e., Petri dishes) of each soil sample (*n =* 3) were created at 10^−3^ and 10^−4^ dilutions. The Petri dishes (9 cm diameter) were incubated at 25 °C for 4–7 days. Subsequently, total colony forming units (CFU) of fungi were quantified and morphological identification at the genus level was performed [26,27], eventually expressing the results as CFU/g dry soil. The isolates unidentified by morphological means were analyzed by means of polymerase chain reaction (PCR) sequencing of amplicons of the internal transcribed spacer region (ITS) rDNA using the primers ITS4 and ITS5 [28] and subsequent database searches using BLASTN software, based on the consensus sequences created by aligning the forward and reverse sequences of the target isolates. The PCR conditions were 5 min at 94 °C, 35 cycles of 1 min at 94 °C, 1 min at 55 °C, 2 min at 72 °C, and a final elongation of 7 min at 72 °C. Purified PCR products were sequenced using the Sanger sequencing method (Instituto de Biología Molecular y Celular de Plantas, Valencia, Spain) and edited via base calling on the BioEdit program.

#### 2.5.2. Assessment of Fungal Microbiota through DNA Metabarcoding

- Soil DNA Isolation and Quantification

DNA from soil samples (*n =* 3) was isolated using the DNeasy PowerSoil Pro DNA isolation kit (Qiagen, Hilden, Germany), strictly following the manufacturer’s instructions. An extraction blank for cross-contamination was included. DNA was quantified using the Qubit High Sensitivity dsDNA Assay (Thermo Fisher Scientific, Waltham, MA, USA).

- Library Preparation and Sequencing

For fungal library preparation, a fragment of the ITS2 genomic region (of about 350 bp) was amplified using the primers ITS86F (5′ GTG AAT CAT CGA ATC TTT GAA 3′) [29] and ITS4R (5′ TCC TCC GCT TAT TGA TAT GC 3′) [28]. Illumina sequencing primers were attached to these primers at their 5′ ends.

PCRs were carried out in a final volume of 12.5 μL, containing 2.5 μL of template DNA, 0.5 μM of primers, 6.25 μL of Supreme NZYTaq 2× Green Master Mix (NZYTech, Lisbon, Portugal), 1× CES [30], and ultrapure water at a volume up to 12.5 μL. The reaction mixture was incubated as follows: an initial denaturation step at 95 °C for 5 min, followed by 35 cycles of 95 °C for 30 s, 49 °C for 45 s, 72 °C for 45 s, and a final extension step at 72 °C for 7 min. A negative control that contained no DNA (BPCR) was included in every PCR round to check for contamination during library preparation. The libraries were run on a 2% agarose gel stained with GreenSafe (NZYTech) and imaged under UV light to verify the library size. Libraries were purified using the Mag-Bind RXNPure Plus magnetic beads (Omega Biotek, Beijing, China), following the instructions provided by the manufacturer. Then, libraries were pooled in equimolar amounts according to the quantification data provided by the Qubit dsDNA HS Assay (Thermo Fisher Scientific). This pool also contained a testimonial amount (1 μL) of the PCR blank and DNA extraction blank (Bex). The pool was sequenced in the MiSeq PE300 run (Illumina). DNA metabarcoding analyses were carried out by AllGenetics & Biology S.L. (La Coruña, Spain; www.allgenetics.eu).

- Quality Control and Processing of Sequencing Data

Illumina paired-end raw forward (R1) and reverse (R2) FASTQ reads were stored in separate files. The quality of the FASTQ files was assessed with the software (v0.11.9) FastQC [31] and the output was summarized using MultiQC [32]. The obtained amplicon reads were processed using QIIME 2 (release 2022.2) [33]. Specifically, the tool DADA2 [34] (implemented in QIIME 2) was used to remove the PCR primers, filter low-quality reads, denoise, merge the forward and reverse reads, remove the chimeric reads, and cluster the resulting sequences into amplicon sequence variants (ASVs). Cutadapt [35] was firstly used to remove primer and/or adapter sequences. The resulting output of the DADA2 pipeline was a table containing the number of occurrences of every observed ASV in each sample. The taxonomic assignment of ASVs was conducted using a pre-trained classifier of the UNITE reference database [36] (updated in May 2021). Specifically, the feature-classifier classify-sklearn approach implemented in QIIME 2 [37] was employed. The following ASVs were excluded: singletons (i.e., ASVs containing only one member sequence in the whole dataset), ASVs occurring at a frequency below 0.01%, unassigned sequences, and sequences assigned only at the kingdom level. The final filtered ASV table was converted into a Biological Observation Matrix (biom) file that was directly imported into R 3.6.1 (R-Team-Core, 2019) using the package phyloseq 1.24.2 [38]. The final filtered ASV and the biom file were used for further analysis and plotting.

#### 2.5.3. Fungal Community Description and Data Procesing

Four classical indexes of biodiversity were selected to describe the structure of the fungal community of each sample: Margalef genus richness (d) [39]; the Shannon–Wiener diversity index (H′) log basis [40]; Pielou’s evenness (J) [41] of the distribution of individuals among genera; and Simpson’s dominance (D) [42]. All calculations were carried out with the software PRIMER version 6.0 (Primer-E Ltd. 239 Plymouth, UK) for Windows [43]. Principal coordinate analysis (PCoA) was used to visualize the potential differences in the soil fungal community structure between treatments or within treatments. Fungal community data (at the genus and operational taxonomic unit (OTU) levels, for plate culture and DNA metabarcoding evaluation methods, respectively) were compared between treatments using permutational analysis of variance (PERMANOVA). This test calculates Pseudo-F statistics to obtain *p*-values [44]. All PERMANOVA tests used 9999 permutations from unrestricted permutations of raw data. Where there were fewer than 99 unique permutations for a meaningful test, approximate Monte Carlo *p*-values (*p*(MC)) were obtained from an asymptotic permutation distribution [45]. Similarity percentage (SIMPER) analysis was used to calculate the average contribution of the abundance of individual culturable fungal genera and OTU to the average dissimilarity of the soil fungal community of the two treatments. For all PERMANOVA and SIMPER analyses, data were square root transformed. The calculation of similarity matrices in the PERMANOVA analysis was based on Bray–Curtis distance. PCoA, PERMANOVA, and SIMPER analyses were performed using the multivariate statistical software package PRIMER-6 with the PERMANOVA+ extension (Primer-E Ltd., Plymouth, UK) for Windows.

The linear discriminant analysis effect size (LEfSe) algorithm [46] was used to identify and compare unique fungal taxa significantly related to any of the two treatments (i.e., fertigation and water) for the different seasons. The threshold for the logarithmic linear discriminant analysis (LDA) score was set at 2.0 and the Wilcoxon *p*-value was set at 0.05. LEfSe was conducted for the results obtained through the two evaluation methods (plate culture method and DNA metabarcoding method).

The FUNGuild database [47] (accessed on 22 March 2023) was used to classify fungal ASVs into functional groups. The FUNGuild software annotates taxonomic data within the OTU table with corresponding data on its online database. The annotations include the guild, trophic mode, and growth morphology, and only confidence scores of “Probable” and “Highly Probable” were considered. The ASVs with confidence scores of “Possible” and those that ended up without functional assignment were placed into the “Unclassified” category.

### 2.6. Tomato Crop Analyzed Variables

#### 2.6.1. Tomato Crop Production

The production was measured for all of the harvests in the 6 experimental plots (2 treatments with 3 replications each). In Year 1, the first harvest was undertaken on 27 December 2019 (101 days after planting (DAP)) and the last was performed on 25 March 2020 (191 DAP). In Year 2, the first harvest was on 30 December 2020 (96 DAP) and the last was performed on 13 April 2021 (200 DAP). In total, 14 and 15 harvests were undertaken in Year 1 and Year 2, respectively. The cumulative tomato production was measured (kg m^−2^) using an electronic balance with an accuracy of ±0.01 kg.

#### 2.6.2. Tomato Fruit Quality

**Figure 1:**
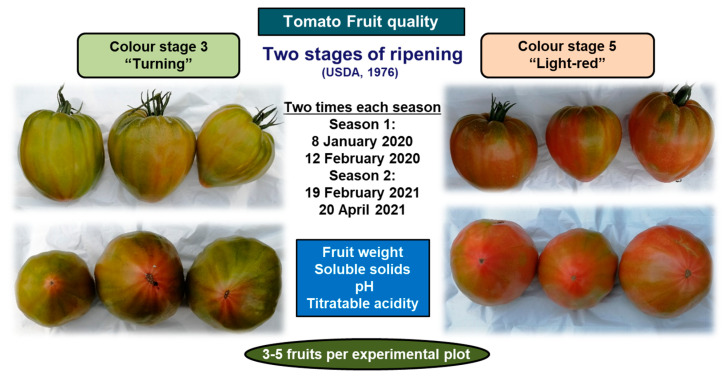
Information about the tomato fruit quality attributes assessed.

The attributes of tomato fruit quality were measured at 2 different times during each crop cycle (i.e., Season 1 and Season 2): on 8 January 2020 and 12 February 2020 in Season 1, and on 19 February 2021 and 20 April 2021 in Season 2. Three and five fruits, in Season 1 and Season 2, respectively, were taken from each experimental plot and harvested at two color stages according to the USDA ripening classes of tomatoes [48,49]: (i) color stage 3 “Turning”, over 10% but not more than 30% red, pink, or orange-yellow and (ii) color stage 5 “Light red”, over 60% but not more than 90% red (Figure 1). The attributes evaluated were as follows. The content of soluble solids (°Brix) was determined with a Smart-1 refractometer (ATAGO, Tokyo, Japen) to ± 0.1 °Brix. Fruit pH and titratable acidity (TA) were determined using a pH electrode and an 862 Compac Titrosampler (Metrohm, Herisau, Switzerland) with accuracy ± 0.01. The TA was expressed as % citric acid after titration with NaOH 0.1 N [50].

### 2.7. Cost of Fertilizers Applied in Fertigated Plots

To estimate the amount of fertilizers used during the tomato crop cycles and determine the economic cost, the fertilization plan applied to the “fertigation” treatment was documented for the two crop cycles considered in this study. The cost per unit of area was calculated based on the consumption of each fertilizer and the price paid to the supplying company for the fertilizers.

### 2.8. Statistical Analysis

Student’s *t*-test was used to determine statistically significant effects and differences among treatments (two levels: fertigation and water) for each of the variables evaluated in the study (physical and chemical variables of soil, soil fungi variables, tomato crop production, and fruit quality variables) for both years. Previously, normality and homoscedasticity were tested using the Shapiro–Wilk and Levene tests, respectively. The Kruskal–Wallis one-way non-parametric test (*p* = 0.05) was performed when normality or homoscedasticity of data was not evident (*p* < 0.05 Shapiro–Wilk or Levene tests, respectively). Arcsine square root transformation was applied to percentages before analyses. These statistical analyses were carried out using the statistical software package Statgraphics Centurion XIX (Statgraphics Technologies, Inc., The Plains, VA, USA) for Windows (Microsoft Corporation, Redmond, WA, USA).

## 3. Results

### 3.1. Physical-Chemical Characteristics of Soil Samples

Out of all of the physical and chemical variables evaluated, only differences in Mg^+2^ concentration (*p* < 0.01, Table 3) were reported at the end of Season 2, being higher in the soil of plots irrigated only with water during the crop season compared to in the soil of those that were fertigated. Overall, the organic fertigation program did not increase any of the physical and chemical variables of soil samples.

### 3.2. Soil Fungi in Soil Samples

#### 3.2.1. Effects of the Treatments on Soil Fungal Diversity Alpha Diversity of Soil Fungal Community

According to the plate culture evaluation method (culturable fungal microbiota), the initial state of the soil before starting the treatments showed an average CFU of 1800 (standard deviation, s.d.: 2779) (Table 4). By the end of Season 1, the average number of CFU was 37,333 and 24,867 in the “fertigation” and “water” soil plots, respectively, while at the end of Season 2, the CFU numbers were 6667 and 10,467, respectively. In all cases, no statistical differences (*p* > 0.05) were detected between the two groups. There were also no differences in the number of identified genera at the end of both seasons, with average values ranging from 5.7 to 6.3, while at the beginning of the study, an average value of 1.7 (s.d.: 1.5) was identified. Additionally, there was no evidence that the fertigation treatment, which used fertilizers listed in Commission Implementing Regulation (EU) 2021/1165, had an impact on the alpha diversity indices of soil culturable fungi (Table 4).

According to the molecular evaluation method based on DNA metabarcoding, at the beginning of the study, the average sequencing depth for the ITS library was 49,649 valid reads (s.d.: 3307) after quality trimming (Table 4). By the end of Season 1, the average number of valid reads was 54,212 and 49,679 in the “fertigation” and “water” soil plots, respectively, while at the end of Season 2, the number of valid reads was 90,645 and 93,058, respectively. In all cases, no statistical differences (*p* > 0.05) were detected between the two groups. There were also no differences in the number of OTUs at the end of both seasons, with average values ranging from 22.0 to 56.6, while at the beginning of the study, an average of 41.3 (s.d.: 8.3) was identified. Additionally, there was no evidence that the fertigation treatment, which used fertilizers listed in Commission Implementing Regulation (EU) 2021/1165, had an impact on the alpha diversity of soil fungi DNA (Table 4).

#### 3.2.2. Comparison of Soil Fungal Community Structure between the Two Treatments Beta Diversity of Soil Fungal Community

**Figure 2:**
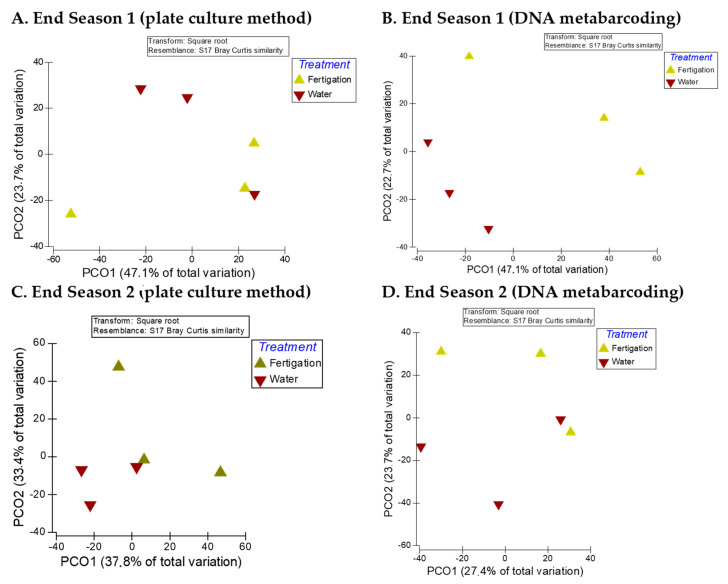
PCoA of the influence of the “water” and “fertigation” treatments on the beta diversity of soil culturable fungi at the genus level and on OTUs identified through DNA metabarcoding at the end of the two crop seasons. (**A**,**C**) Plate culture method (culturable fungi); (**B**,**D**) DNA metabarcoding (OTUs).

The results of PCoA showed that the fungal communities in the samples of the two treatments had relatively discrete distribution at the end of the two seasons regardless of the analytical method (Figure 2), with relatively large distances between the samples of the two treatments. This is more evident with data from DNA metabarcoding analysis and mainly at the end of Season 1 (Figure 2B), since at the end of Season 2 (Figure 2D), two samples, one from each treatment, are located relatively close to each other.

According to SIMPER analysis, the fungal community composition identified at the genera level through the plate culture evaluation method (culturable fungal microbiota) in the fertigation and water treatments showed an average dissimilarity of 63.98 and 61.12 at the end of Season 1 and Season 2, respectively (Table 5). The contribution percentage of each culturable fungus genus to the dissimilarity between two treatments (water and fertigation), as well as the cumulative contribution percentage of that genus, is shown in Table 5. The table displays the 13 and 12 genera that were identified at the end of Season 1 and Season 2, respectively, along with their average abundance (note that data were square root transformed) in both the fertigation and water treatments. Although their contribution is different in each season, *Acremonium*, *Penicillium*, *Mortierella*, and *Aspergillus* are the top four genera that contribute the most to the dissimilarity between water and fertigation at the end of both seasons. These top four genera contributed to over 65% of the dissimilarity between the two treatments.

On the other hand, the fungal community composition identified at the OTU level through DNA metabarcoding in the fertigation and water treatments showed an average dissimilarity of 79.46 and 76.43 at the end of Season 1 and Season 2, respectively (Table 6). The 30 main OTUs that contributed the most to the differences in the microbial community between soils irrigated with water and through the fertigation program during the tomato crop cycles at the end of the two crop seasons contributed to over 92% and 93% of the dissimilarity between the two treatments at the end of Season 1 and Season 2, respectively. At the end of Season 1, the values range from 0.65% to 14.88%, with the most dissimilar OTU being from the phylum *Ascomycota*. The second and third most dissimilar OTUs are from the phylum *Rozellomycota* and the genus *Plectosphaerella*, respectively. It is worth noting that these three OTUs together contribute to more than 42% of the dissimilarity observed between the two groups. This is followed by the phylum *Basidiomycota* and the genus *Aspergillus*, both contributing more than 4%. At the end of Season 2, the results show that again the phylum *Ascomycota* had the highest average abundance in both treatments, and the highest contribution to the differences between the two treatments (14.18%). The second and third highest contributors were the fungal genera *Aspergillus* (7.81%) and *Thermomyces* (6.66%), respectively. This is followed by the phylum *Rozellomycota* and the genus *Mortierella*, with a contribution of more than 6% in both cases. These five OTUs contribute to more than 41% of the dissimilarity observed between the two groups at the end of Season 2.

However, PERMANOVA analysis showed that the composition of the fungal communities did not differ significantly between the treatments at the end of the two seasons, neither for cultivable fungal communities (end of Season 1: Pseudo-F = 0.7579, *p*(MC) = 0.5431; end of Season 2: Pseudo-F = 1.5156, *p*(MC) = 0.2561) nor for fungal communities identified through DNA metabarcoding (end of Season 1: Pseudo-F = 2.161, *p*(MC) = 0.1409; end of Season 2: Pseudo-F = 1.0015, *p*(MC) = 0.4346).

#### 3.2.3. Analysis of Differences in Fungal Genera Dominance

**Figure 3:**
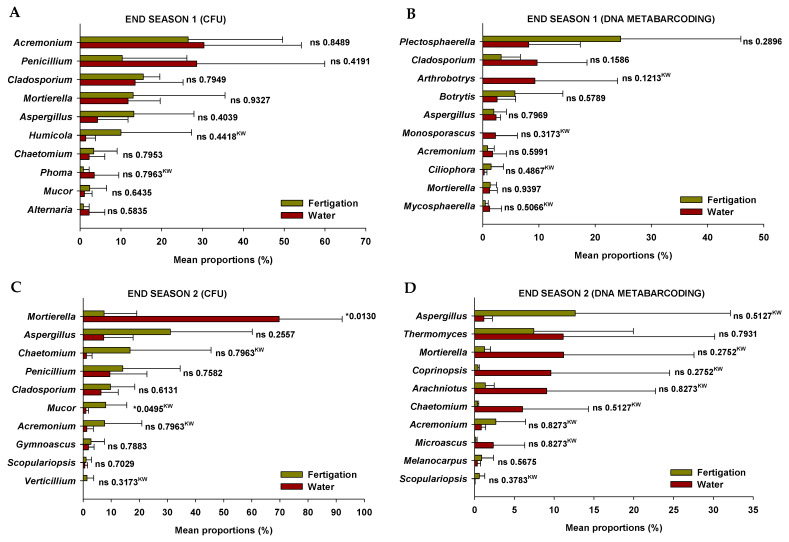
Relative abundance of fungal genera detected in soil through the two analytical methods at the end of the two crop seasons in plots irrigated with water and through the organic fertigation program during the tomato crop cycles. (**A**,**C**) Plate culture method (CFU); (**B**,**D**) DNA metabarcoding. Values (mean ± standard deviation; *n* = 3) are shown for the two analytical methods performed in the study of soil fungal microbiota (plate culture method and DNA metabarcoding). ‘*’ indicates significance at *p* ≤ 0.05 (Student’s *t*-test or Kruskal–Wallis test). ‘ns’ indicates non significance. ‘^KW^’ *p*-value through the Kruskal–Wallis non-parametric test.

The relative abundance of the ten most abundant fungal genera detected in soil through the two analytical methods (plate culture method and DNA metabarcoding) at the end of the two crop seasons in plots irrigated with water and through the organic fertigation program during the tomato crop cycles is shown in Figure 3. Significant differences were only exhibited for two cultivable fungal genera at the end of Season 2 (Figure 3C), while in no case were differences exhibited in the relative abundance of fungal genera identified through DNA metabarcoding (Figure 3B,C). The cultivable fungal genera that showed differences were *Mortierella*, which exhibited higher relative abundance in the “water” treatment soils, and *Mucor*, which was more abundant in the “fertigation” treatment soils. These differences do not appear to be linked to the inoculum of cultivable fungal genera incorporated through irrigation in both treatments (Figure S1).

#### 3.2.4. Taxonomic Groups Associated to Fertilization Practices

**Figure 4:**
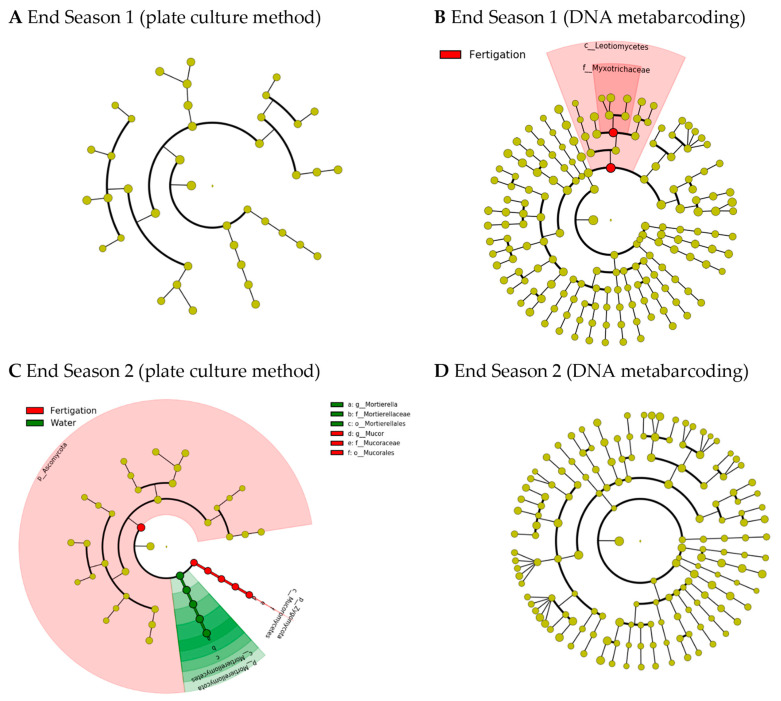
LEfSe cladogram analysis of the species differential abundance detected in soil through the culture medium analytical method ((**A**), end Season 1; (**C**), end Season 2) and DNA metabarcoding ((**B**), end Season 1; (**D**), end Season 2) in fungal communities after the plots were irrigated with water (“water”) and through the organic fertigation program (“fertigation”) during the tomato crop cycles. LDA scores greater than 2.0. Note: No biomarkers were identified at the end of Season 1 through the culture medium analytical method or at the end of Season 2 through the DNA metabarcoding analytical method.

LEfSe analysis identified biomarkers that caused significant differences between the two treatments at the end of the two seasons, although they did not match between the two analytical methods (Figure 4). Thus, at the end of Season 1, only two biomarkers were identified in the soil fungal community analyzed by means of DNA metabarcoding in the “fertigation” treatment (Figure 4B) with taxa at different taxonomic levels (the class *Leotiomycetes* and the family *Myxotrichaceae*). At the end of Season 1, no biomarkers were detected in the cultivable soil fungi genera (Figure 4A). However, contrary to the previous results, at the end of Season 2, only two biomarkers were detected in the cultivable soil fungi genera (Figure 4C), but no biomarkers were detected in the soil fungal community analyzed by means of DNA metabarcoding (Figure 4D). In this case, the genus *Mortierella* was detected as a biomarker of the “water” treatment, while the phylum *Ascomycota* and the genus *Mucor* was detected as a biomarker of the “fertigation” treatment. All identified biomarkers had LDA scores greater than 5.

#### 3.2.5. Nutritional and Functional Analyses of Fungal Communities identified through DNA Metabarcoding

FUNGuild was used to predict the nutritional and functional groups of the fungal communities with different treatments that were identified through DNA metabarcoding at the end of the two seasons. At the end of Season 1, a total of 97 ASVs were identified, of which 43.3% did not receive functional assignment and 6.2% received “Possible” confidence scores, so the results are presented for 50.5% of the identified ASVs. This represents 53.8% (s.d. 16.8) and 39.7% (s.d. 10.5) of the total “fertigation” and “water” sequencing reads, respectively. At the end of Season 2, a total of 197 ASVs were identified, of which 48.2% did not receive functional assignment and 12.7% received “Possible” confidence scores, so the results are presented for 39.1% of the identified ASVs. This represents 28.6% (s.d. 35.9) and 33.2% (s.d. 27.0) of the total “fertigation” and “water” sequencing reads, respectively.

**Figure 5:**
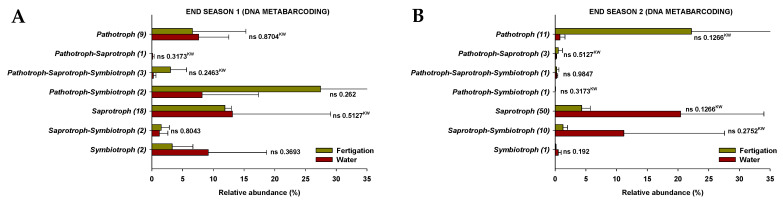
The relative abundance of trophic modes assigned by FUNGuild for fungal communities at the end of Season 1 (**A**) and Season 2 (**B**). ‘ns’ indicates non significance at *p* ≤ 0.05 (Student’s *t*-test or Kruskal–Wallis test). ‘^KW^’ *p*-value through the Kruskal–Wallis non-parametric test.

**Figure 6:**
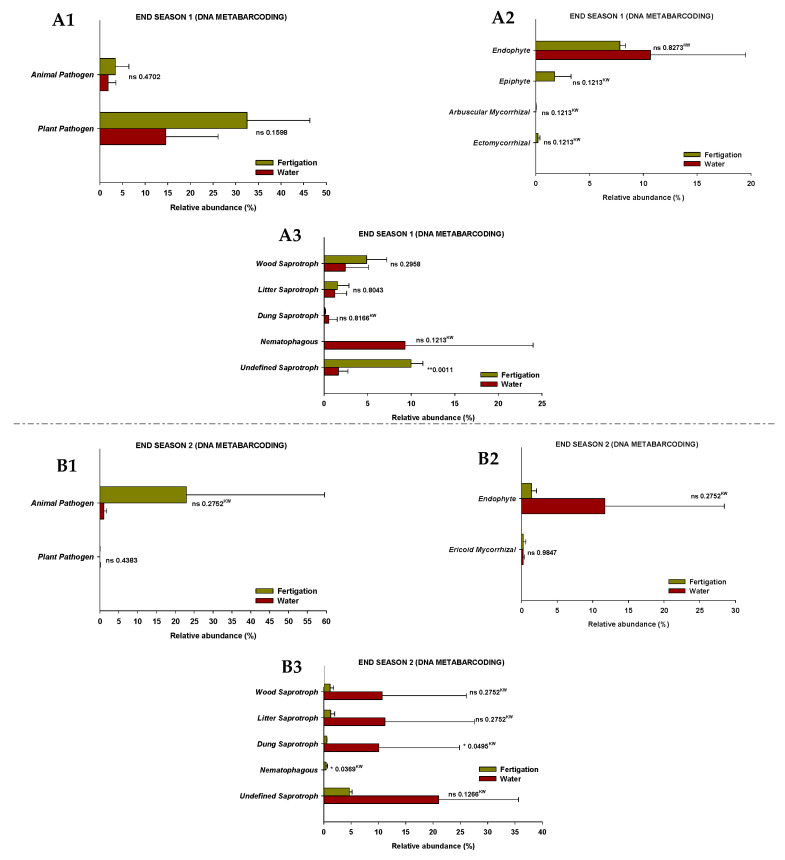
The relative abundance of guilds assigned by FUNGuild for fungal communities at the end of Season 1 (**A1**–**A3**) and Season 2 (**B1**–**B3**). (1) Pathotroph trophic mode, (2) symbiotroph trophic mode, (3) saprotroph trophic mode. ‘*’ and ‘**’ indicates significance at *p* ≤ 0.05 and *p* ≤ 0.01 (Student’s *t*-test or Kruskal–Wallis test), respectively. ‘ns’ indicates non significance. ‘^KW^’ *p*-value through the Kruskal–Wallis non-parametric test.

The results show that the fungi community was divided into seven trophic mode groups. The fungal community was screened for 39 and 47 identifiable species, at the end of Season 1 and Season 2, respectively, to which FUNGuild assigned confidence scores of “Probable” and “Highly Probable”. They included saprotrophs (46.2% and 57.4% at the end of Season 1 and Season 2, respectively), pathotrophs (23.1% and 17.0%), pathotroph-symbiotrophs (10.3% and 2.1%), pathotroph-saprotroph-symbiotrophs (10.3% and 2.1%), saprotroph-symbiotrophs (5.1% and 12.8%), pathotroph-saprotrophs (2.6% and 6.4%), and symbiotrophs (5.1% and 2.1%), representing the general abundance of each nutrition method in the identified community (Figure 5). Among them, pathotrophs, saprotrophs, and symbiotrophs were the major components, so the species with various trophic modes assigned were considered for all of them. The pathotrophic mode groups primarily consisted of plant pathogens (not representing a specific specie) and animal pathogens, which showed different relative abundances depending on the season, but no differences were identified between treatments (Figure 6(A1,B1)).

In terms of symbiotrophic mode groups, at the end of Season 1, endophytes, epiphytes, arbuscular mycorrhizae, and ectomycorrhizae were identified, while endophytes and ericoid mycorrhizae were identified at the end of Season 2. However, no significant differences in the relative abundances were found between treatments (Figure 6(A2,B2)). The mode groups of saprotrophs included undefined saprotrophs, dung saprotrophs, litter saprotrophs, wood saprotrophs, and nematophagous fungi. The relative abundance of undefined saprotrophs and nematophagous fungi was higher (*p* < 0.05) in the “fertigation” treatment compared to the “water” treatment at the end of Season 1 and Season 2, respectively. Nevertheless, the relative abundance of dung saprotrophs was higher (*p* < 0.05) in the “water” treatment at the end of Season 2 (Figure 6(A3,B3)).

### 3.3. Tomato Crop

#### 3.3.1. Tomato Crop Production

**Figure 7:**
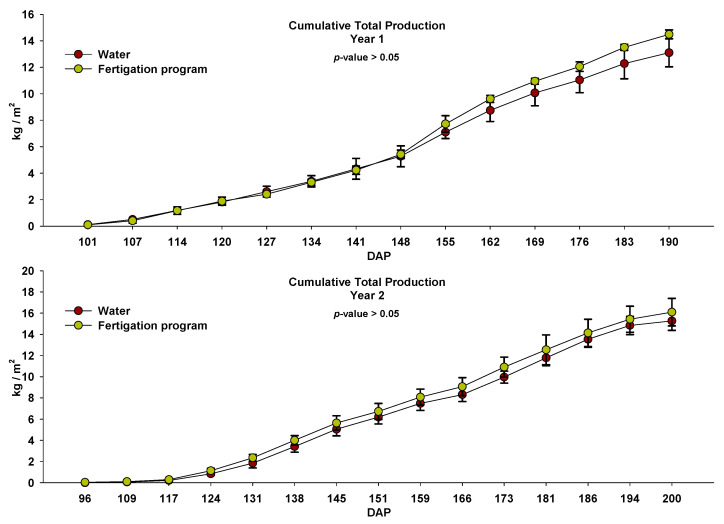
Cumulative tomato production in the two years of study in plots irrigated with water and through the fertigation program during the crop cycles. Values (mean ± standard deviation; *n* = 3). DAP: days after planting.

There were no significant differences between the two treatments in any of the harvests nor in the accumulated marketable tomato production in either of the two years of study (Figure 7). In plots that only received basal dressing with fresh sheep manure (i.e., “Water” treatment), the total marketable tomato production was 13.10 and 15.27 kg m^−2^ in Season 1 and Season 2, respectively. In plots that also received nutrient inputs through fertigation (i.e., “fertigation” treatment), the production was 14.49 and 16.09 kg m^−2^ in Season 1 and Season 2, respectively. The higher production obtained in the second year was mainly due to a longer crop cycle than the first.

#### 3.3.2. Tomato Fruit Quality

Only the Brix degrees of the tomatoes at the “Turning” color stage showed significant differences between treatments in one of the sampling times, being higher in those from the fertigation treatment compared to those from the water treatment (Table 7). In this case, the higher EC of the irrigation water from the fertigation treatment (Table S1) may have influenced the Brix degrees of the fruits evaluated. None of the rest of the tomato fruit quality variables tested showed significant differences between treatments.

### 3.4. Cost of Fertilizers Applied in the Two Treatments

The cost of fertilizers applied during the crop cycles in the fertigated plots (Table 2), along with the corresponding cost of the sheep manure incorporated prior to the start of the crop seasons (i.e., EUR 4200.0 ha^−1^, not including labor), amounted to EUR 17,768.7 and EUR 24,245.7 ha^−1^ (including VAT) in Season 1 and Season 2, respectively. The incorporation of sheep manure as a basal dressing without subsequent fertigation resulted in a cost savings of 76.4–82.7% compared to the cost of organic fertigated plots.

## 4. Discussion

The sustainability of agricultural systems relies on the type and intensity of the cultural practices carried out. Basal dressing fertilization and continuous fertigation during the crop season are common practices for vegetable growers in intensive systems, including greenhouse organic farming. Both types of fertilization, using organic matter, can influence crop profitability and soil fertility, including its impact on soil fungal communities. In the present study, the incidence and impact of continued fertigation with fertilizers listed in the Commission Implementing Regulation (EU) 2021/1165 on products and substances for use in organic production on the main physical and chemical variables of soil and its fungal communities, as well as on the production and quality of greenhouse tomato fruit, were evaluated for two consecutive years after a basal fresh sheep manure application. To obtain a more detailed understanding of the impact on soil fungal communities, the study was conducted using both plate culture (culturable fungi) and DNA metabarcoding methods. Additionally, a study of the cost of fertilizers applied during the two crop cycles in the fertigated plots was conducted. At the end of the two crop cycles, none of the physicochemical variables were increased in the fertigated plots compared to those where soils only received water during the crop cycles and the only added fertilizer was the basal fresh sheep manure. Concerning soil fungal communities, there were no significant differences between the treatments in the alpha diversity indices of the soil fungal community or in the fungal community composition, and differences were only found regarding the identification of biomarkers. The fertilizers added through fertigation did not consistently improve the tomato yield and fruit quality in any of the two years studied, despite their high cost.

In terms of soil physicochemical properties, in this study, we only detected differences in the exchangeable magnesium (Mg^+2^) content at the end of the second year. It was found in higher concentrations in the soils irrigated with water during the tomato crop cycles, which had been previously amended with fresh sheep manure. These differences could be influenced by the extraction of this fraction of the mineral element from the soil by the metabolic products of a living organism. For instance, the importance of Mg in the metabolism of fungi in the *Aspergillus* genus (e.g., *A. niger*) has been widely reported, and several authors have successfully demonstrated the ability of the fungi to extract Mg from the soil for its own growth [51,52,53]. In this regard, a technique based on the correlation between the weight of mycelium of the fungus and the available magnesium content of the soil has been successfully used [52,53]. In our study, although we did not detect significant differences in the relative abundance of *Aspergillus* in soils due to the treatments, this fungal genus was identified through the two analytical methods as one of the main OTUs and/or genera that contributed the most to the differences in the microbial community between soils irrigated with water and those fertigated during the tomato crop cycles at the end of the two crop seasons. The average abundance of *Aspergillus* was higher in the fertigated soils; thus, the extraction of Mg^+2^ could also be higher, which could be the reason why the content of Mg^+2^ in the soil was lower. This is only a possible hypothesis, and it should be noted that while the role of soil fungi, such as mycorrhizae, in N and P uptake and balance has been intensively studied, the effects on metal nutrients such as Mg are less clear and more inconsistent in cultivated soils for vegetable crops [54].

The availability of an adequate nutrient supply is crucial for ensuring optimal growth and productivity in plants [55]. Inadequate synchronization between nutrient availability and plant uptake has been recognized as a significant limitation to the productivity of organic plant production systems [56,57]. In our study, the basal fresh sheep manure application was sufficient for the correct development of a greenhouse tomato crop, without showing any deficiencies due to a lack of nutrient availability during the two-year study period. In this sense, the incorporated manure could have promoted the prevalence of soil microbial communities, including the fungal community, capable of meeting the crop’s needs throughout the growing season. In this regard, while standardized methods for evaluating soil physicochemical parameters have been in place for several decades, the same cannot be said for the study of fungal microbiota in soil. Continuous efforts have been devoted to the standardization of the numerous methods that have been developed over the last few decades to enable a better understanding of the structure and ecology of soil microbial communities and their roles in soil functioning [58,59,60]. In this respect, the emergence of high-throughput sequencing (including DNA metabarcoding) in recent years has allowed scientists worldwide to identify soil fungi. The majority of published studies utilize Illumina MiSeq to characterize fungi, with a focus on targeting ITS regions, especially ITS2, which is also considered the optimal technique for comparative studies [10]. On the other hand, the plate culture method makes it possible to detect and identify culturable fungal species. This method is increasingly less widely used mainly because it is laborious, time-consuming, and requires the expertise of taxonomists for accurate identification. In addition, the microbial diversity of soil microbiome is highly underestimated, as it is estimated that only 1–5% of total soil microorganisms have been cultured by current methodologies [61,62]. However, while through high-throughput DNA sequencing both the live (including active and inactive fractions) and dead fungi are identified, the culturable fungi identified from a soil sample are considered active or potentially active fungal groups. The latter exhibit specific functions or trophic requirements with a certain degree of certainty, allowing for studies on their functionality [61]. In our study, we used both methods for studying the soil mycobiome. Using the plate culture method, a total of 13 and 12 fungal genera were identified at the end of Seasons 1 and 2, respectively, resulting in a total of 16 different fungal genera identified throughout the entire study. Through DNA metabarcoding, 40 and 50 fungal genera were identified at the end of Seasons 1 and 2, respectively, resulting in a total of 74 different fungal genera identified throughout the entire study. Although the results we obtained must be considered complementary, for the purpose of comparing the evaluated treatments in relation to soil fungal communities, both techniques led us to the same conclusions. Thus, the analysis of fungi alpha diversity showed no differences in any of the calculated indices due to the treatments, regardless of the evaluation method for soil fungi assessment. Similarly, the PERMANOVA analysis did not show significant differences in the composition of the fungal communities (fungal beta diversity), although the SIMPER analyses showed high dissimilarity values that were reflected in the PCoA plots. Nevertheless, the LEfSe analysis identified biomarkers that caused significant differences between the two treatments at the end of the two seasons, although they did not match between the two analytical methods. The class *Leotiomycetes* and the family *Myxotrichaceae* (included in the class *Leotiomycetes*) were identified as biomarkers in the soil fungal community analyzed by means of DNA metabarcoding of the “fertigation” treatment at the end of Season 1. The genus *Mortierella* was detected as a biomarker of the “water” treatment, and *Mucor* was identified as a biomarker of the “fertigation” treatment in the culturable soil fungi at the end of Season 2. *Leotiomycetes* (inoperculate discomycetes) is a class of *Ascomycota* which includes powdery mildews that are classified in the order *Erysiphales* and the family *Erysiphaceae* [63]. Likewise, it includes the family *Myxotrichaceae*, a cellulolytic group that encompasses several species. In this regard, *Myxotrichum deflexum* was exclusively identified in the ‘fertigation’ treatment at the end of Season 1. *Gymnascella nodulosa*, on the other hand, was identified in both treatments but only at the end of Season 2. Additionally, *Oidiodendron periconioides* and *Oidiodendron echinulatum*, which are *Oidiodendron* anamorphs, were exclusively identified in the ‘fertigation’ treatment at the end of Season 1. Moreover, *Pseudogymnoascus roseus* was exclusively identified in the ‘fertigation’ treatment at the end of Season 1. Lastly, *Gymnascella dankaliensis*, a *Gymnoascus* anamorph, was identified in both treatments but only at the end of Season 1. These are fungal species that have been shown to be able to form typical ericoid mycorrhizas [64]. Ericoid mycorrhizal fungi have saprotrophic and biotrophic lifestyles and are able to biodegrade SOM and establish symbiotic relationships with plants in the family *Ericaceae* [65]. Therefore, these types of mycorrhizae are not classified as beneficial for tomato plants, which belong to the *Solanaceae* family. Furthermore, this result was not consistent after two years of treatments (i.e., end of Season 2). Meanwhile, *Mortierella* is a fungal genus known for its ability to decompose organic matter and participate in nutrient cycles in the soil, and some species of fungi in the *Mortierella* genus demonstrate the ability to solubilize phosphorus (P) [66]. Recently, the work of [67] revealed that substituting manure for mineral P fertilizer significantly increases P availability in greenhouse soil. The authors suggested that this could largely be attributed to the enrichment of P-mineralizing microbes and indicated that manure is a feasible substitute for mineral P fertilizers in greenhouse farming. In addition, species from the *Mortierella* genus have also been recognized as plant-growth-promoting fungi [68] and as potential antagonistic agents against various plant pathogens [69,70,71]. On the other hand, the *Mucor* genus is a fungal genus classified in the *Mucorales*, the most prominent order of zygospore-forming fungi that formerly constituted the *Zygomycota*, a phylum which is not currently accepted due to polyphyly. While *Mucor* is primarily a saprobic fungal genus, some species have been identified as plant endophytes, and others have been found to be opportunistic pathogens of animals and humans. It is worth noting that no species of *Mucor* have been reported to have mutualistic associations with plants [72].

Our results also show a significant presence of fungi from other fungal genera, such as *Acremonium*, *Aspergillus*, and *Penicillium*, which were identified through the two analytical methods and were present at all sampling time points regardless of the treatment. These fungi have been recognized as phosphorus solubilizers [73,74,75,76,77] as well as plant-growth-promoting fungi [76,78,79,80]. In order to obtain additional information, in the present study, the FUNGuild database was used to predict the nutritional and functional groups of fungal communities identified through DNA metabarcoding. No differences were identified between treatments in both pathotrophic and symbiotrophic modes, but differences were observed in the saprotrophic mode. Specifically, the relative abundance of undefined saprotrophs and nematophagous fungi was significantly higher in the “fertigation” treatment compared to the “water” treatment at the end of Season 1 and Season 2, respectively. However, the relative abundance of dung saprotrophs was higher in the “water” treatment at the end of Season 2. When interpreting these results, it is crucial to exercise caution due to their limited consistency, as they were not consistently observed throughout all sampling time points. Additionally, their low representativeness should be taken into account, considering the relatively small proportion of sequencing reads they encompass. Additional in-depth studies on soil fungal functional groups are also needed to further investigate the function of the soil fungal community as a function of the type of sustainable fertilization and to obtain a more comprehensive understanding of the role of fungi in soil agroecosystems. On the other hand, an issue that caught our attention is that while *Exophiala* appeared as the fungal genus with the highest relative abundance both in the water and in the fertilizer solution applied in the plots, this yeast was not identified in the soils by any of the evaluation methods in any case. Soil microbial communities are highly complex and diverse and are typically resistant to invasion by applied microorganisms [81,82].

Numerous studies have evaluated the impact of fertilizers on soil fertility, including the soil fungal community. In recent years, there have been several studies comparing the impacts of conventional fertilizers (i.e., chemical synthetic fertilizers) versus organic fertilizers [20,83,84,85,86,87]. There are also studies comparing different organic fertilizers applied as basal amendments before crop installation, organic fertilizers applied through fertigation, or both in combination [21,86], and even studies comparing combinations of chemical fertilizers with organic fertilizers [88,89,90,91,92]. According to these studies, these practices generally have a significant impact on soil fungal communities. For instance, several studies have demonstrated that the use of inorganic fertilizers leads to a decrease in both fungal diversity and biomass [84,93]. Conversely, the application of organic fertilizers can alter the composition and abundance of fungal communities, potentially resulting in an increase in fungal diversity in some cases [93,94]. However, there have been reports of an increase in fungal diversity when inorganic fertilizers were applied, both with and without basal manure application [86,87,90]. In addition, the organic fertilizer type and composition seem to be relevant for determining the diversity of soil fungi communities [91]. Thus, a recent meta-analysis of 37 studies on the effect of organic and mineral fertilizers on soil microbial diversity suggests that a large amount of further research is required to fully understand the influence of fertilizer regimes on microbial diversity and ecosystem function [83]. Therefore, the extent of the impact on soil fungal communities varies, and it remains uncertain whether results obtained from studies conducted on specific soils and agroecosystems can be used to infer trends at a broader scale. Furthermore, irrigation and fertilization levels have also shown significant effects on fungal community diversity [95].

To the best of our knowledge, no research to date has examined the impact of organic fertilizers incorporated through fertigation following basal fresh sheep manure application on soil fertility, including the soil fungal community, and on tomato crop yield in intensive greenhouse vegetable production systems. This practice has a significant impact on the sustainability and profitability of organic vegetable production systems in greenhouses, where improving soil health and reducing agricultural inputs are essential. Thus, this study is equally relevant to farmers who decide to initiate the necessary conversion process to certify their greenhouse area as “organic” according to Regulation (EU) No 2018/848 on organic production. The study presented here was conducted for two consecutive years, the required timeframe for horticultural crops. According to the obtained results, the continued organic fertigation after basal manure application does not impact soil fungal communities, tomato yield, or soil fertility. Therefore, incorporating manure as a basal fertilizer in intensive tomato crops is proposed as a crucial practice to achieve more sustainable and profitable greenhouse agriculture. Consequently, it emerges as a key practice to overcome the primary challenge of shifting soil modeling from a chemical-based approach to an agroecological approach [4], and thus enables most farmers to make this transition.

## Figures and Tables

**Table 1:** Treatments considered in the study.

Treatments	
Water	Sheep manure at a rate of 4 kg m^−2^. Irrigated solely with water throughout the crop cycle.
Fertigation	Sheep manure at a rate of 4 kg m^−2^ + a fertilization plan using fertilizers listed in the Commission Implementing Regulation (EU) 2021/1165 on products and substances for use in organic production which was applied through fertigation throughout tomato crop cycle (Table 2).

**Table 2:** Fertilizers applied throughout the two tomato crop cycles (Season 1 and Season 2) on the organic fertigation plots, including their cost.

Fertilizers	Price (kg or L)	Amount (kg ha^−1^ or L ha^−1^)		Cost (€ ha^−1^ VAT Included 10%)	
		Season 1	Season 2	Season 1	Season 2
Acetic acid	0.67	1413.33	3883.33	1041.62	2862.01
Calcium	2.40	850.00	1150.00	2244.00	3036.00
Magnesium sulphate	0.38	453.33	493.33	189.49	206.21
Potassium sulphate	0.79	1683.33	1933.33	1462.81	1680.06
Sodium chloride	0.24	198.00	833.33	52.25	220.00
Fertiliser 4-1-7.5 + 0.5 Mg	3.50	1533.33	2208.33	5903.32	8502.07
Microelements	2.90	113.33	182.67	361.52	582.18
Amino acids	2.35	666.67	766.67	1723.34	1981.84
Humic acids	1.40	383.33	633.33	590.33	975.33
Total Cost (VAT included)				13,568.69	20,045.70

**Table 3:** Soil physical and chemical variables at the beginning of the study (start of Season 1), and at the end of the two growing seasons, in plots irrigated with water and through the fertigation program during the tomato crop cycles. Values (mean ± standard deviation; *n* = 3).

Soil Physical and Chemical Variables	Start Season 1	End Season 1			Soil Physical and Chemical Variables	End Season 2		
		Fertigation	Water	*p*-Value		Fertigation	Water	*p*-Value
pH	8.1 ± 0.4	7.9 ± 0.1	8.1 ± 0.2	0.196	pH (Extract 1:2:5 H_2_O)	9.1 ± 0.1	9.0 ± 0.1	0.101
CE (dS/m) *	7.05 ± 4.24	1.18 ± 0.17	1.35 ± 0.60	0.652	CE (dS/m) **	0.39 ± 0.03	0.31 ± 0.06	0.103
Ca^+2^ (mg/L)	1714 ± 525	3667 ± 1494	4427 ± 898	0.497	Ca^+2^ (mg/kg) ^y^	5679 ± 72	5807 ± 85	0.117
Na^+1^ (mg/L)	36 ± 7	54 ± 15	42 ± 5	0.268	Na^+1^ (mg/kg) ^y^	134 ± 20	116 ± 39	0.513
Mg^+2^ (mg/L)	303 ± 120	343 ± 37	420 ± 86	0.226	Mg^+2^ (mg/kg) ^y^	238 ± 5	291 ± 19	**0.009**
K^+1^ (mg/L)	1000 ± 289	1945 ± 860	742 ± 293	0.084	K^+1^ (mg/kg) ^y^	576 ± 33	420 ± 169	0.192
Active limestone (%)	6.8 ± 2.8	5.2 ± 1.0	6.5 ± 1.0	0.204	Active limestone (%) ^y^	8.0 ± 1.0	7.7 ± 0.6	0.682
P Olsen (meq/L)	26 ± 3	9 ± 3	7 ± 1	0.483	P Olsen (mg/kg) ^y^	41.3 ± 3.6	47.9 ± 6.5	0.200
Nitric N (mg/L)	239 ± 183	30 ± 8	32 ± 11	0.874	Nitric N (mg/kg) ^y^	7.2 ± 4.5	8.2 ± 1.0	0.711
Organic Matter (%)	1.4 ± 0.0	1.2 ± 0.2	0.9 ± 0.1	0.093	Organic Matter (%) ^y^	1.1 ± 0.1	1.3 ± 0.2	0.563
Total carbonates (%)	12 ± 3	18 ± 4	24 ± 3	0.106	CaCO_3_ equivalent (%) ^y^	29 ± 3	27 ± 2	0.304
Total N (%)	0.043 ± 0.006	0.043 ± 0.006	0.037 ± 0.006	0.237	Total N (%) ^y^	0.110 ± 0.010	0.120 ± 0.010	0.288
C/N	22 ± 3	18 ± 2	17 ± 1	0.292	C/N	6.0 ± 0.1	6.1 ± 0.7	0.766

* Results in saturated extract at 25 °C. ** Results in extract 1:5 H_2_O at 25 °C. ^y^ Results expressed on dry weight basis, bold *p*-values denote statistical differences through the Student’s *t*-test.

**Table 4:** Alpha diversity indices of soil fungal community at the beginning of the study (start of Season 1), and at the end of the two crop seasons, in plots irrigated with water and through the organic fertigation program during the tomato crop cycles. Values (mean ± standard deviation; *n* = 3) are shown for the two analytical methods performed in the study of soil fungal microbiota (plate culture method and DNA metabarcoding).

Plate Culture Method							
Soil Fungal Community Alpha Diversity Indices	Start Season 1	End Season 1			End Season 2		
		Fertigation	Water	*p*-Value *	Fertigation	Water	*p*-Value *
No. of CFU	1800.0 ± 2778.5	37,333.3 ± 40,808.5	24,866.7 ± 32,327.3	0.700	6666.7 ± 4875.8	10,466.7 ± 5064.9	0.402
No. of genera	1.7 ± 1.5	6.0 ± 1.7	6.3 ± 0.6	0.768	5.7 ± 0.6	6.3 ± 2.3	0.653
Richness—Margalef (*d*)	0.20 ± 0.05	0.51 ± 0.11	0.57 ± 0.05	0.453	0.54 ± 0.05	0.58 ± 0.24	0.789
Diversity—Shannon (*H*’. log_e_)	0.49 ± 0.43	1.44 ± 0.06	1.40 ± 0.16	0.683	1.32 ± 0.24	0.94 ± 0.49	0.285
Evenness—Pielou (*J*)	0.86 ± 0.20	0.83 ± 0.12	0.76 ± 0.12	0.542	0.77 ± 0.15	0.50 ± 0.16	0.100
Dominance—Simpson (*D*)	0.47 ± 0.04	0.72 ± 0.04	0.66 ± 0.10	0.398	0.87 ± 0.01	0.79 ± 0.08	0.144
**DNA Metabarcoding**							
**Soil Fungal Community** **Alpha Diversity Indices**	**Start Season 1**	**End Season 1**			**End Season 2**		
		**Fertigation**	**Water**	*p*-Value *	**Fertigation**	**Water**	*p*-Value *
Reads	49,649.0 ± 3306.6	54,212.3 ± 6450.8	49,679.3 ± 2727.7	0.325	90,645.3 ± 5955.1	93,058.3 ± 8211.3	0.701
OTUs	41.3 ± 8.3	43.3 ± 21.1	22.0 ± 4.6	0.162	56.6 ± 5.5	55.3 ± 3.8	0.7471
Richness—Margalef (*d*)	3.90 ± 0.59	5.00 ± 2.20	2.72 ± 0.61	0.158	5.27 ± 0.41	5.21 ± 0.51	0.884
Diversity—Shannon (*H*′. log_e_)	2.21 ± 0.21	2.62 ± 0.22	2.15 ± 0.37	0.129	1.85 ± 0.47	2.12 ± 0.38	0.477
Evenness—Pielou (*J*)	0.60 ± 0.07	0.72 ± 0.07	0.70 ± 0.09	0.745	0.46 ± 0.11	0.53 ± 0.10	0.437
Dominance—Simpson (*D*)	0.83 ± 0.03	0.87 ± 0.01	0.79 ± 0.08	0.144	0.64 ± 0.17	0.74 ± 0.16	0.473

* p-values through the Student’s *t*-test.

**Table 5:** Contribution (Contrib%) and cumulative contribution (Cum%) of the SIMPER analysis of all of the fungal genera (culturable fungi) identified through the plate culture method that contributed to the differences in the microbial community between soils irrigated with water and through the fertigation program during the tomato crop cycles at the end of the two crop seasons.

End Season 1 (Average Dissimilarity = 63.98)					End Season 2 (Average Dissimilarity = 61.12)				
	Fertigation	Water				Fertigation	Water		
Genus	Av.Abund	Av.Abund	Contrib%	Cum.%	Genus	Av.Abund	Av.Abund	Contrib%	Cum.%
*Acremonium*	94.25	49.06	19.76	19.76	*Mortierella*	15.25	81.99	31.48	31.48
*Penicillium*	44.72	83.85	18.03	37.79	*Aspergillus*	45.25	22.30	15.46	46.94
*Mortierella*	59.63	35.72	15.98	53.77	*Penicillium*	22.31	22.35	10.74	57.68
*Aspergillus*	37.98	29.81	11.34	65.10	*Acremonium*	17.64	4.71	8.34	66.03
*Cladosporium*	61.08	41.91	8.49	73.59	*Chaetomium*	12.91	6.67	7.71	73.74
*Mucor*	14.91	14.91	4.83	78.42	*Cladosporium*	15.62	24.34	7.65	81.39
*Phoma*	14.91	10.54	4.56	82.98	*Mucor*	18.83	9.43	5.04	86.43
*Humicola*	8.16	6.67	3.82	86.79	*Gymnoascus*	5.27	9.43	3.93	90.36
*Alternaria*	14.91	4.71	3.78	90.58	*Scopulariopsis*	6.67	4.71	3.44	93.8
*Calcarisporium*	0.00	14.91	2.71	93.29	*Verticillium*	4.71	0.00	2.24	96.05
*Fusarium*	14.91	0.00	2.47	95.76	*Fusarium*	0.00	4.71	2.12	98.17
*Simplicillium*	4.71	0.00	2.26	98.02	*Alternaria*	0.00	4.71	1.83	100.00
*Chaetomium*	4.71	4.71	1.98	100.00					

Note: The average abundance (Av.abund) refers to the number of CFU, which was square root transformed.

**Table 6:** Contribution (Contrib%) and cumulative contribution (Cum%) of the SIMPER analysis of the 30 main OTUs identified through DNA metabarcoding that contributed the most to the differences in the microbial community between soils irrigated with water and through the fertigation program during the tomato crop cycles at the end of the two crop seasons.

End Season 1 (Average Dissimilarity = 79.46)					End Season 2 (Average Dissimilarity = 79.43)				
	Fertigation	Water				Fertigation	Water		
OTU	Av.Abund	Av.Abund	Contrib%	Cum.%	OTU	Av.Abund	Av.Abund	Contrib%	Cum.%
*p__Ascomycota*	39.47	40.34	14.88	14.88	*p__Ascomycota*	152.49	70.36	14.18	14.18
*p__Rozellomycota*	60.43	13.67	14.05	28.93	*g__Aspergillus*	103.36	31.54	7.81	21.99
*g__Plectosphaerella*	60.26	18.07	13.59	42.52	*g__Thermomyces*	44.97	51.17	6.66	28.65
*p__Basidiomycota*	9.97	7.06	4.51	47.03	*p__Rozellomycota*	83.24	47.02	6.62	35.27
*g__Aspergillus*	11.23	8.98	4.25	51.28	*g__Mortierella*	33.82	63.05	6.06	41.33
*g__Blastobotrys*	16.13	0.00	3.30	54.58	*g__Arachniotus*	27.87	83.12	5.68	47.01
*g__Trichoderma*	14.18	0.00	2.90	57.48	*o__Hypocreales*	39.37	62.29	5.53	52.54
*g__Arthrobotrys*	0.00	10.07	2.88	60.36	*g__Amesia*	64.53	9.39	4.96	57.50
*g__Oidiodendron*	13.73	0.00	2.81	63.17	*g__Chaetomium*	16.06	64.52	4.44	61.94
*o__Hypocreales*	2.79	5.73	2.45	65.62	*p__Basidiomycota*	29.53	21.40	3.75	65.69
*g__Cladosporium*	9.32	14.65	2.40	68.02	*f__Chaetomiaceae*	8.95	56.11	3.66	69.35
*g__Ciliophora*	6.56	1.49	2.07	70.09	*g__Coprinopsis*	12.5	53.34	3.48	72.83
*g__Mortierella*	9.49	4.75	1.95	72.04	*g__Acremonium*	35.28	17.93	2.56	75.39
*g__Botrytis*	7.53	5.98	1.76	73.80	*f__Plectosphaerellaceae*	32.07	11.01	2.52	77.91
*g__Penicillium*	5.84	1.73	1.69	75.49	*g__Microascus*	7.19	24.08	1.93	79.84
*g__Candida*	7.80	0.00	1.60	77.09	*g__Sporendonema*	6.57	20.36	1.85	81.69
*f__Pseudeurotiaceae*	7.42	0.00	1.52	78.61	*f__Nectriaceae*	0.00	18.36	1.48	83.17
*g__Scytalidium*	6.71	0.00	1.37	79.98	*g__Wallemia*	18.69	3.05	1.44	84.61
*c__Sordariomycetes*	0.00	4.63	1.34	81.32	*o__Sordariales*	10.85	3.47	1.12	85.73
*g__Geminibasidium*	3.79	0.00	1.25	82.57	*g__Scopulariopsis*	12.46	2.60	0.96	86.69
*g__Myxotrichum*	3.73	0.00	1.23	83.80	*g__Arthrobotrys*	12.29	0.00	0.94	87.63
*o__Helotiales*	5.86	0.00	1.20	85.00	*g__Remersonia*	0.00	11.41	0.92	88.55
*g__Monosporascus*	0.00	4.32	1.18	86.18	*g__Melanocarpus*	12.40	7.81	0.91	89.46
*g__Mycosphaerella*	3.46	3.00	1.13	87.31	*g__Polypaecilum*	11.70	0.00	0.81	90.27
*g__Acremonium*	3.96	4.67	0.96	88.27	*g__Cladosporium*	4.37	9.79	0.65	90.92
*g__Saitozyma*	4.55	0.00	0.93	89.20	*g__Mycothermus*	7.91	0.00	0.63	91.55
*g__Wardomycopsis*	3.06	1.56	0.83	90.03	*g__Ciliophora*	5.30	3.79	0.54	92.09
*f__Didymellaceae*	0.00	2.92	0.76	90.79	*g__Penicillium*	1.76	4.81	0.52	92.61
*g__Chaetomium*	0.00	2.60	0.75	91.54	*g__Gymnascella*	6.18	8.29	0.50	93.11
*g__Meliniomyces*	3.17	0.00	0.65	92.19	*g__Nigrosabulum*	3.57	5.59	0.48	93.59

Note: The contributions of OTUs with the same taxonomic classification are added, thus considering them as a single OTU. In addition, the OTUs that were identified at the species taxonomic level are grouped into their respective genera. The average abundance (Av.abund) refers to the number of reads, which was square root transformed.

**Table 7:** Tomato fruit quality variables in plots irrigated with water and through the organic fertigation program during the tomato crop cycles. Assessments performed at two different times during each crop cycle (i.e., Season 1 and Season 2). Values (mean ± standard deviation; *n* = 3 in Season 1 and *n =* 5 in Season 2).

	Fruit Quality Attributes				
Treatment	Fruit Weight (g)	Soluble Solids (°Brix)	Active Acidity (pH)	Titratable Acidity (% Citric Acid)	Colour Stage ^x^
Season 1					
8 January 2020					
Water	--	3.58 ± 0.13	3.87 ± 0.09	0.46 ± 0.05	3 “Turning”
Fertigation	--	3.51 ± 0.18	3.94 ± 0.05	0.43 ± 0.04	
*p*-value	--	0.596	0.309	0.445	
Water	--	3.71 ± 0.25	3.88 ± 0.04	0.47 ± 0.01	5 “Light-red”
Fertigation	--	3.61 ± 0.18	3.92 ± 0.09	0.43 ± 0.05	
*p*-value	--	0.576	0.505	0.291	
12 February 2020					
Water	313.4 ± 16.7	3.97 ± 0.16	4.62 ± 0.05	0.39 ± 0.04	3 “Turning”
Fertigation	380.5 ± 76.0	4.21 ± 0.12	4.68 ± 0.08	0.33 ± 0.05	
*p*-value	0.210	0.104	0.339	0.207	
Water	525.5 ± 85.1	4.23 ± 0.22	4.54 ± 0.03	0.38 ± 0.04	5 “Light-red”
Fertigation	489.1 ± 137.6	4.38 ± 0.18	4.60 ± 0.04	0.36 ± 0.04	
*p*-value	0.717	0.447	0.101	0.510	
Season 2					
19 February 2021					
Water	358.2 ± 19.6	3.55 ± 0.15	4.43 ± 0.11	0.50 ± 0.06	3 “Turning”
Fertigation	356.8 ± 13.3	3.86 ± 0.10	4.35 ± 0.08	0.53 ± 0.06	
*p*-value	0.9211	**0.044**	0.375	0.662	
Water	375.4 ± 24.8	4.32 ± 0.34	4.39 ± 0.18	0.45 ± 0.04	5 “Light-red”
Fertigation	340.2 ± 42.7	4.40 ± 0.34	4.40 ± 0.05	0.46 ± 0.08	
*p*-value	0.2836	0.799	0.908	0.902	
20 April 2021					
Water	242.5 ± 37.6	4.32 ± 0.13	4.03 ± 0.10	0.50 ± 0.02	3 “Turning”
Fertigation	232.6 ± 42.1	4.52 ± 0.04	4.03 ± 0.08	0.56 ± 0.08	
*p*-value	0.7747	0.072	0.964	0.275	
Water	268.2 ± 23.6	4.77 ± 0.09	3.13 ± 0.09	0.75 ± 0.25	5 “Light-red”
Fertigation	266.2 ± 29.8	4.69 ± 0.16	3.67 ± 0.35	0.60 ± 0.02	
*p*-value	0.9318	0.447	0.061	0.375	

Bold *p*-values denote statistical differences through the Student’s *t*-test. ^x^ According to USDA ripening classes of tomatoes [48,49].

## Data Availability

The data presented in this study are available within the article and Supplementary Materials.

## Supplementary Materials

The following supporting information can be downloaded at: https://www.mdpi.com/article/10.3390/microorganisms11071715/s1, Figure S1: Culturable fungal genera identified in water and fertigation solution applied in the experimental plots. Samples taken on 25 February 2021. Values (mean ± standard deviation; *n* = 3). ‘*’ indicates significance at *p* ≤ 0.05 (Student’s *t*-test or Kruskal–Wallis test), respectively. ‘ns’ indicates non significance. ‘x’ *p*-value through the Kruskal–Wallis non-parametric test; Table S1: Electrical conductivity (EC) of irrigation water (“water”) and nutrient solution supplied through fertigation (“fertigation”) in the experimental plots. M1: sample taken on 25 February 2021; M2: sample taken on 3 March 2021.

## References

[1] (2023). Eurostat. https://ec.europa.eu/eurostat/statistics-explained/index.php?title=Organic_farming_statistics#Total_organic_area.

[2] Junta de Andalucía (2022). Producción Ecológica en Andalucía Balance 2021. https://www.juntadeandalucia.es/export/drupaljda/DECO22_BalancePE21_.pdf.

[3] Cajamar (2022). Análisis de la Campaña Hortofrutícola de Almería. Campaña 2021/2022. https://publicacionescajamar.es/series-tematicas/informes-coyuntura-analisis-de-campana/analisis-de-la-campana-hortofruticola-de-almeria-campana-2021-2022.

[4] Giagnocavo C., de Cara-García M., González M., Juan M., Marín-Guirao J.I., Mehrabi S., Rodríguez E., van der Blom J., Crisol-Martínez E. (2022). Reconnecting Farmers with Nature through Agroecological Transitions: Interacting Niches and Experimentation and the Role of Agricultural Knowledge and Innovation Systems. Agriculture.

[5] Diacono M., Montemurro F. (2010). Long-term effects of organic amendments on soil fertility. A review. Agric. Sustain. Dev..

[6] Gamliel A., van Bruggen A.H.C. (2016). Maintaining soil health for crop production in organic greenhouses. Sci. Hortic..

[7] Pankhurst C.E., Lynch J.M. (1995). The role of soil microbiology in sustainable intensive agriculture. Adv. Plant Pathol..

[8] Tikhonovich I.A., Provorov N.A. (2011). Microbiology is the basis of sustainable agriculture: An opinion. Ann. Appl. Biol..

[9] Ellouze W., Taheri A.E., Bainard L.D., Yang C., Bazghaleh N., Navarro-Borrell A., Hanson K., Hamel C. (2014). Soil fungal resources in annual cropping systems and their potential for management. Biomed. Res. Int..

[10] Frąc M., Hannula E.S., Bełka M., Salles J.F., Jedryczka M. (2022). Soil mycobiome in sustainable agriculture. Front. Microbiol..

[11] Schlatter D., Kinkel L., Thomashow L., Weller D., Paulitz T. (2017). Disease Suppressive Soils: New Insights from the Soil Microbiome. Phytopathology.

[12] Marín-Guirao J.I., De Cara-García M., Crisol-Martínez E., Gómez-Tenorio M.Á., García-Raya P., Tello-Marquina J.C. (2019). Association of plant development to organic matter and fungal presence in soils of horticultural crops. Ann. Appl. Biol..

[13] Camacho-Sanchez M., Herencia J.F., Arroyo F.T., Capote N. (2023). Soil Microbial Community Responses to Different Management Strategies in Almond Crop. J. Fungi.

[14] Hartman K., van der Heijden M.G.A., Wittwer R.A., Banerjee S., Walser J.-C., Schlaeppi K. (2018). Cropping practices manipulate abundance patterns of root and soil microbiome members paving the way to smart farming. Microbiome.

[15] Hartmann M., Frey B., Mayer J., Mäder P., Widmer F. (2014). Distinct soil microbial diversity under long-term organic and conventional farming. ISME J..

[16] Larkin R.P., Honeycutt C.W., Griffin T.S. (2006). Effect of swine and dairy manure amendments on microbial communities in three soils as influenced by environmental conditions. Biol. Fertil. Soils.

[17] Morugán-Coronado A., Pérez-Rodríguez P., Insolia E., Soto-Gómez D., Fernández-Calviño D., Zornoza R. (2022). The impact of crop diversification, tillage and fertilization type on soil total microbial, fungal and bacterial abundance: A worldwide meta-analysis of agricultural sites. Agric. Ecosyst. Environ..

[18] Pérez-Piqueres A., Edel-Hermann V., Alabouvette C., Steinberg C. (2006). Response of soil microbial communities to compost amendments. Soil Biol. Biochem..

[19] Schutter M.E., Sandeno J.M., Dick R.P. (2001). Seasonal, soil type, and alternative management influences on microbial communities of vegetable cropping systems. Biol. Fertil. Soils.

[20] Windisch S., Sommermann L., Babin D., Chowdhury S.P., Grosch R., Moradtalab N., Walker F., Höglinger B., El-Hasan A., Armbruster W. (2021). Impact of Long-Term Organic and Mineral Fertilization on Rhizosphere Metabolites, Root–Microbial Interactions and Plant Health of Lettuce. Front. Microbiol..

[21] Wu J., Shi Z., Zhu J., Cao A., Fang W., Yan D., Wang Q., Li Y. (2022). Taxonomic response of bacterial and fungal populations to biofertilizers applied to soil or substrate in greenhouse-grown cucumber. Sci. Rep..

[22] Valera D.L., Belmonte L.J., Molina-Aiz F.D., López A., Camacho F. (2017). The greenhouses of Almería, Spain: Technological analysis and profitability. Acta Hortic..

[23] Baeza E., Pérez-Parra J., López J.C., Gázquez J.C., Sánchez-Guerrero M.C., Lorenzo P., Alonso F.J., Medrano E. (2010). Ventilación natural. Manejo del Clima en el Invernadero Mediterráneo.

[24] (1976). BOE num. 78. España, Orden de 5 de diciembre de 1975 por la que se aprueban como oficiales los métodos de análisis de suelos y aguas. Boletín Of. Del Estado.

[25] Tello J.C., Vares F., Lacasa A. (1991). Análisis de muestras. Manual de Laboratorio. Diagnóstico de Hongos. Bacterias y Nematodos Fitopatógenos.

[26] Barnett H.L., Hunter B.B. (1972). Illustrated Genera of Imperfect Fungi.

[27] Ellis M.B., Commonwealth Mycological Institute (1971). Dematiaceous Hyphomycetes.

[28] White T.J., Bruns T., Lee S., Taylor J., Innis M.A. (1990). Amplification and direct sequencing of fungal ribosomal RNA genes for phylogenetics. PCR Protocols. A Guide to Methods and Applications.

[29] Turenne C.Y., Sanche S.E., Hoban D.J., Karlowsky J.A., Kabani A.M. (1999). Rapid Identification of Fungi by Using the ITS2 Genetic Region and an Automated Fluorescent Capillary Electrophoresis System. J. Clin. Microbiol..

[30] Ralser M., Querfurth R., Warnatz H.-J., Lehrach H., Yaspo M.-L., Krobitsch S. (2006). An Efficient and Economic Enhancer Mix for Pcr. Biochem. Biophys. Res. Commun..

[31] Andrews S. (2010). FastQC: A Quality Control Tool for High Throughput Sequence Data.

[32] Ewels P., Magnusson M., Lundin S., Käller M. (2016). MultiQC: Summarize analysis results for multiple tools and samples in a single report. Bioinformatics.

[33] Bolyen E., Rideout J.R., Dillon M.R., Bokulich N.A., Abnet C.C., Al-Ghalith G.A., Alexander H., Alm E.J., Arumugam M., Asnicar F. (2019). Reproducible, interactive, scalable and extensible microbiome data science using QIIME 2. Nat. Biotechnol..

[34] Callahan B.J., McMurdie P.J., Rosen M.J., Han A.W., Johnson A.J.A., Holmes S.P. (2016). DADA2: High-resolution sample inference from Illumina amplicon data. Nat. Methods.

[35] Martin M. (2011). Cutadapt removes adapter sequences from high-throughput sequencing reads. EMBnet J..

[36] Abarenkov K., Zirk A., Piirmann T., Pöhönen R., Ivanov F., Nilsson R.H., Kõljalg U. (2020). UNITE QIIME release for Fungi. UNITE Community.

[37] Bokulich N.A., Kaehler B.D., Rideout J.R., Dillon M., Bolyen E., Knight R., Huttley G.A., Caporaso J.G. (2018). Optimizing taxonomic classification of marker-gene amplicon sequences with QIIME 22019s q2-feature-classifier plugin. Microbiome.

[38] McMurdie P.J., Holmes S. (2013). phyloseq: An R Package for Reproducible Interactive Analysis and Graphics of Microbiome Census Data. PLoS ONE.

[39] Margalef D.R. (1958). Information Theory in Ecology. Gen. Syst..

[40] Shannon C.E., Weaver W. (1949). The Mathematical Theory of Communication.

[41] Pielou E.C. (1969). An Introduction to Mathematical Ecology.

[42] Simpson E.H. (1949). Measurement of Diversity. Nature.

[43] Clarke K.R., Gorley R.N. (2006). Primer v6: User Manual or Tutorial.

[44] Anderson M.J. (2001). A new method for non-parametric multivariate analysis of variance. Austral Ecol..

[45] Anderson M.J., Robinson J. (2003). Generalized discriminant analysis based on distances. Aust. NZ J. Stat..

[46] Afgan E., Baker D., Batut B., Van Den Beek M., Bouvier D., Ech M., Chilton J., Clements D., Coraor N., Grüning B.A. (2018). The galaxy platform for accessible, reproducible and collaborative biomedical analyses: 2018 update. Nucleic Acids Res..

[47] Nguyen N.H., Song Z., Bates S., Branco S., Tedersoo L., Menke J., Schilling J., Kennedy P. (2016). FUNGuild: An open annotation tool for parsing fungal community datasets by ecological guild. Fungal Ecol..

[48] Gierson D., Kader A.A., Atherton J.G., Rudich J. (1986). Fruit ripening and quality. The Tomato Crop.

[49] USDA (1976). United States Standards for Grade of Fresh Tomatoes.

[50] AOAC (1984). Official Methods of Analysis.

[51] Nicholas D.J.D., Fielding A.H. (1951). The Use of Aspergillus Niger (M)* for the Determination of Magnesium, Zinc, Copper and Molybdenum Available in Soils to Crop Plants. J. Hortic. Sci..

[52] Henriksen A. (1972). Biologically available versus exchangeable magnesium in soil. Plant Soil.

[53] Metson A.J.I. (1974). Some factors governing the availability of soil magnesium: A review. N. Z. J. Exp. Agric..

[54] Sardans J., Lambers H., Preece C., Alrefaei A.F., Penuelas J. (2023). Role of mycorrhizas and root exudates in plant uptake of soil nutrients (calcium, iron, magnesium, and potassium): Has the puzzle been completely solved?. Plant J..

[55] Ingestad T., Ågren G.I. (1995). Plant nutrition and growth: Basic principles. Plant Soil.

[56] Berry P.M., Sylvester-Bradley R., Philipps L., Hatch D.J., Cuttle S.P., Rayns F.W., Gosling P. (2002). Is the productivity of organic farms restricted by the supply of available nitrogen?. Soil Use Manag..

[57] Nygaard J., Thorup-Kristensen K. (2011). Plant-based fertilizers for organic vegetable production. J. Plant Nutr. Soil Sci..

[58] Philippot L., Ritz K., Pandard P., Hallin S., Martin-Laurent F. (2012). Standardisation of methods in soil microbiology: Progress and challenges. FEMS Microbiol. Ecol..

[59] Römbke J., Bernard J., Martin-Laurent F. (2018). Standard methods for the assessment of structural and functional diversity of soil organisms: A review. Integr. Environ. Assess. Manag..

[60] van Gestel C.A., Mommer L., Montanarella L., Pieper S., Coulson M., Toschki A., Rutgers M., Focks A., Römbke J. (2021). Soil Biodiversity: State-of-the-Art and Possible Implementation in Chemical Risk Assessment. Integr. Environ. Assess. Manag..

[61] Blagodatskaya E., Kuzyakov Y. (2013). Active microorganisms in soil: Critical review of estimation criteria and approaches. Soil Biol. Biochem..

[62] Mendes R., Garbeva P., Raaijmakers J.M. (2013). The rhizosphere microbiome: Significance of plant beneficial, plant pathogenic, and human pathogenic microorganisms. FEMS Microbiol. Rev..

[63] Glawe D.A. (2008). The Powdery Mildews: A Review of the World’s Most Familiar (Yet Poorly Known) Plant Pathogens. Annu. Rev. Phytopathol..

[64] Dalpe Y. (1989). Ericoid mycorrhizal fungi in the Myxotrichaceae and Gymnoascaceae. New Phytol..

[65] Wei X., Zhang W., Zulfiqar F., Zhang C., Chen J. (2022). Ericoid mycorrhizal fungi as biostimulants for improving propagation and production of ericaceous plants. Front. Plant Sci..

[66] Sang Y., Jin L., Zhu R., Yu X.-Y., Hu S., Wang B.-T., Ruan H.-H., Jin F.-J., Lee H.-G. (2022). Phosphorus-Solubilizing Capacity of Mortierella Species Isolated from Rhizosphere Soil of a Poplar Plantation. Microorganisms.

[67] Sun R., Niu J., Luo B., Wang X., Li W., Zhang W., Wang F., Zhang C., Ye X. (2022). Substitution of manure for mineral P fertilizers increases P availability by enhancing microbial potential for organic P mineralization in greenhouse soil. Front. Bioeng. Biotechnol..

[68] Ozimek E., Hanaka A. (2021). Mortierella Species as the plant growth-promoting fungi present in the agricultural soils. Agriculture.

[69] Tagawa M., Tamaki H., Manome A., Koyama O., Kamagata Y. (2010). Isolation and characterization of antagonistic fungi against potato scab pathogens from potato field soils. FEMS Microbiol. Lett..

[70] Wills W.H., Lambe R.C. (1980). Mortierella antagonism to oomycetes. Phytopathology.

[71] Xiong W., Li R., Ren Y., Liu C., Zhao Q., Wu H., Jousset A., Shen Q. (2017). Distinct roles for soil fungal and bacterial communities associated with the suppression of vanilla Fusarium wilt disease. Soil Biol. Biochem..

[72] Morin-Sardin S., Nodet P., Coton E., Jean-Luc J. (2017). Mucor: A Janus-faced fungal genus with human health impact and industrial applications. Fungal Biol. Rev..

[73] Ichriani G.I., Syehfani S., Nuraini Y., Handayanto E. (2018). Formulation of Biochar-Compost and Phosphate Solubilizing Fungi from Oil Palm Empty Fruit Bunch to Improve Growth of Maize in an Ultisol of Central Kalimantan. J. Ecol. Eng..

[74] Vassileva M., Mendes G.d.O., Deriu M.A., Benedetto G.d., Flor-Peregrin E., Mocali S., Martos V., Vassilev N. (2022). Fungi, P-Solubilization, and Plant Nutrition. Microorganisms.

[75] Harvey P.R., Warren R.A., Wakelin S. (2009). Potential to improve root access to phosphorus: The role of non-symbiotic microbial inoculants in the rhizosphere. Crop. Pasture Sci..

[76] Sánchez-Esteva S., Gómez-Muñoz B., Jensen L.S., de Neergaard A., Magid J. (2016). The effect of Penicillium bilaii on wheat growth and phosphorus uptake as affected by soil pH, soil P and application of sewage sludge. Chem. Biol. Technol. Agric..

[77] Vera-Morales M., López Medina S.E., Naranjo-Morán J., Quevedo A., Ratti M.F. (2023). Nematophagous Fungi: A Review of Their Phosphorus Solubilization Potential. Microorganisms.

[78] Khan M.S., Gao J., Munir I., Zhang M., Liu Y., Moe T.S., Xue J., Zhang X. (2021). Characterization of Endophytic Fungi, Acremonium sp., from Lilium davidii and Analysis of Its Antifungal and Plant Growth-Promoting Effects. Biomed. Res. Int..

[79] Mundim G.d.S.M., Maciel G.M., Mendes G.d.O. (2022). Aspergillus niger as a Biological Input for Improving Vegetable Seedling Production. Microorganisms.

[80] Yamagiwa Y., Inagaki Y., Ichinose Y., Toyoda K., Hyakumachi M., Shiraishi T. (2011). Talaromyces wortmannii FS2 emits-caryphyllene, which promotes plant growth and induces resistance. J. Gen. Plant Pathol..

[81] Mallon C.A., Poly F., Le Roux X., Marring I., van Elsas J.D., Salles J.F. (2015). Resource pulses can alleviate the biodiversity-invasion relationship in soil microbial communities. Ecology.

[82] O’Callaghan M., Ballard R.A., Wright D. (2022). Soil microbial inoculants for sustainable agriculture: Limitations and opportunities. Soil Use Manag..

[83] Bebber D.P., Richards V.R. (2022). A meta-analysis of the effect of organic and mineral fertilizers on soil microbial diversity. Appl. Soil Ecol..

[84] Lin X., Feng Y., Zhang H., Chen R., Wang J., Zhang J., Chu H. (2012). Long-term balanced fertilization decreases arbuscular mycorrhizal fungal diversity in an arable soil in North China revealed by 454 pyrosequencing. Environ. Sci. Technol..

[85] Yang L.J., Zhao F.Y., Chang Q., Li T.L., Li F.S. (2015). Effects of vermicomposts on tomato yield and quality and soil fertility in greenhouse under different soil water regimes. Agric. Water Manag..

[86] Cuartero J., Özbolat O., Sánchez-Navarro V., Egea-Cortines M., Zornoza R., Canfora L., Orrù L., Pascual J.A., Vivo J.-M., Ros M. (2021). Changes in Bacterial and Fungal Soil Communities in Long-Term Organic Cropping Systems. Agriculture.

[87] Semenov M.V., Krasnov G.S., Semenov V.M., van Bruggen A. (2022). Mineral and Organic Fertilizers Distinctly Affect Fungal Communities in the Crop Rhizosphere. J. Fungi.

[88] Zhao H.T., Li T.P., Zhang Y., Hu J., Bai Y.C., Shan Y.H., Ke F. (2017). Effects of vermicompost amendment as a basal fertilizer on soil properties and cucumber yield and quality under continuous cropping conditions in a greenhouse. J. Soils Sediments.

[89] Solaiman Z.M., Shafi M.I., Beamont E., Anawar H.M. (2020). Poultry Litter Biochar Increases Mycorrhizal Colonisation, Soil Fertility and Cucumber Yield in a Fertigation System on Sandy Soil. Agriculture.

[90] Tao R., Hu B., Chu G. (2020). Impacts of organic fertilization with a drip irrigation system on bacterial and fungal communities in cotton field. Agric. Syst..

[91] Guo X., Liu J., Xu L., Sun F., Ma Y., Yin D., Gao Q., Zheng G., Lv Y. (2022). Combined Organic and Inorganic Fertilization Can Enhance Dry Direct-Seeded Rice Yield by Improving Soil Fungal Community and Structure. Agronomy.

[92] Su Y., Zi H., Wei X., Hu B., Deng X., Chen Y., Jiang Y. (2022). Application of Manure Rather Than Plant-Origin Organic Fertilizers Alters the Fungal Community in Continuous Cropping Tobacco Soil. Front. Microbiol..

[93] Ding J., Jiang X., Guan D., Zhao B., Ma M., Zhou B., Cao F., Yang X., Li L., Li J. (2017). Influence of inorganic fertilizer and organic manure application on fungal communities in a long-term field experiment of Chinese Mollisols. Appl. Soil Ecol..

[94] Xiang X., Liu J., Zhang J., Li D., Xu C., Kuzyakov Y. (2020). Divergence in fungal abundance and community structure between soils under long-term mineral and organic fertilization. Soil Tillage Res..

[95] Ye Z., Li J., Wang J., Zhang C., Liu G., Dong Q. (2021). Diversity and co-occurrence network modularization of bacterial communities determine soil fertility and crop yields in arid fertigation agroecosystems. Biol. Fertil. Soils.

