# Supplementary material for: Continued Organic Fertigation after Basal Manure Application Does Not Impact Soil Fungal Communities, Tomato Yield or Soil Fertility
Source: Microorganisms. 2023 Jun 30;11(7):1715. doi: 10.3390/microorganisms11071715 (PMC10386759; doi:10.3390/microorganisms11071715)
Supplement: Supplementary file 1 [file microorganisms-11-01715-s001.zip › microorganisms-2475000-supplementary.pdf]

Table S1. Electrical conductivity (EC) of irrigation water ("water") and nutrient solution supplied through fertigation ("fertigation") in the experimental plots. M1: sample taken on 25 February 2021; M2: sample taken on 3 March 2021.

	Water	Fertigation	*p*-value [X]
EC M1	0.85±0.02	3.28±0.06	0
EC M2	0.79±0.00	3.32±0.08	0

Bold *p*-values denote statistical differences through the Student's t-test. [X] Statistical analysis performed with the square root transformation.

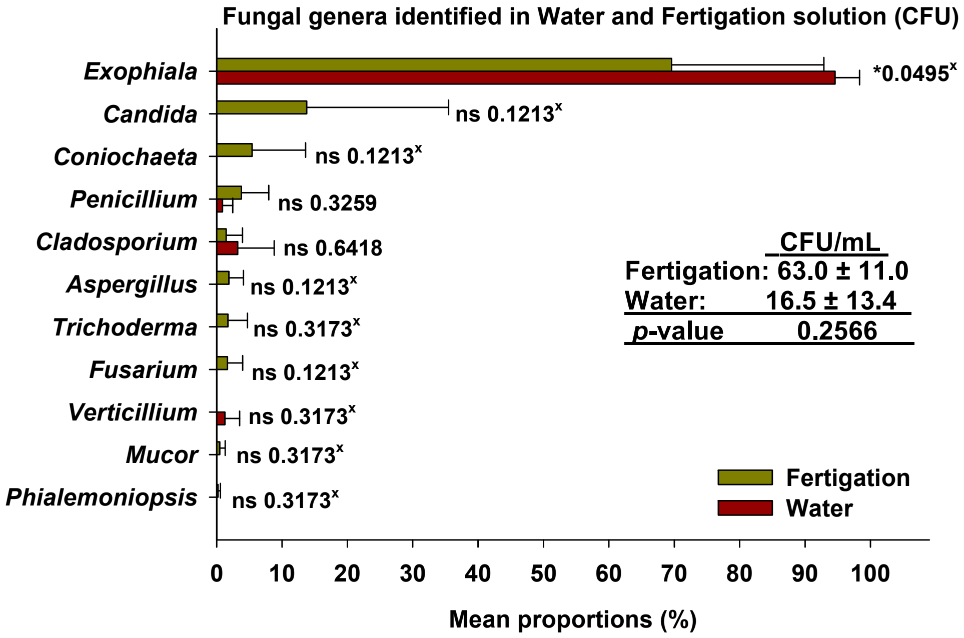

Figure S1. Culturable fungal genera identified in the water and the fertigation solution applied in the experimental plots. Samples taken on 25/02/21. Values (mean ± standard deviation; $n = 3$). '*' indicates significance at $p \leq 0.05$ (t-Student or Kruskal–Wallis test), respectively. 'ns' indicates non significance. 'x' *p*-value through the Kruskal–Wallis non-parametric test.
